# Comparative analysis of methods for gene transcription profiling data derived from different microarray technologies in rat and mouse models of diabetes

**DOI:** 10.1186/1471-2164-10-63

**Published:** 2009-02-05

**Authors:** Steven P Wilder, Pamela J Kaisaki, Karène Argoud, Anita Salhan, Jiannis Ragoussis, Marie-Thérèse Bihoreau, Dominique Gauguier

**Affiliations:** 1 The Wellcome Trust Centre for Human Genetics, University of Oxford, Roosevelt Drive, Headington, Oxford, OX3 7BN, UK

## Abstract

**Background:**

Microarray technologies are widely used to quantify the abundance of transcripts corresponding to thousands of genes. To maximise the robustness of transcriptome results, we have tested the performance and reproducibility of rat and mouse gene expression data obtained with Affymetrix, Illumina and Operon platforms.

**Results:**

We present a thorough analysis of the degree of reproducibility provided by analysing the transcriptomic profile of the same animals of several experimental groups under different popular microarray technologies in different tissues. Concordant results from inter- and intra-platform comparisons were maximised by testing many popular computational methods for generating fold changes and significances and by only considering oligonucleotides giving high expression levels. The choice of Affymetrix signal extraction technique was shown to have the greatest effect on the concordance across platforms. In both species, when choosing optimal methods, the agreement between data generated on the Affymetrix and Illumina was excellent; this was verified using qRT-PCR on a selection of genes present on all platforms.

**Conclusion:**

This study provides an extensive assessment of analytical methods best suited for processing data from different microarray technologies and can assist integration of technologically different gene expression datasets in biological systems.

## Background

Microarray analysis is an established method of investigating the transcription levels of many thousands of genes simultaneously [1]. Several technologies have been developed, differing in array design, manufacturing procedure (standardised printing or randomised microbeads), experimental design (absolute or relative expression level), target oligonucleotide sequence length, hybridisation and image analysis. The most advanced and widely used technologies for high throughput gene transcription studies are Affymetrix^® ^GeneChip^® ^[2] and Illumina^® ^Sentrix^® ^BeadChip arrays [3], which both allow the quantification of the amount of tens of thousands of transcripts. Custom arrays of oligonucleotides have also been used and should add biological information providing that data can be integrated with these more advanced technologies.

On Affymetrix arrays, genes are represented by one or more probe sets, which are short oligonucleotides (25 base pairs) covering distinct sections of the gene synthesised in place through photolithography. Illumina BeadChips^® ^have an entirely unique layout of 50 base pairs long oligonucleotides synthesised in a separate procedure and attached covalently to silica beads, which are randomly dispersed over the array so that they each lie in an unique well [4]. This random allocation of beads means that each oligonucleotide is represented a random number of times on each array (on average 30 times). Custom microarrays consist of long oligonucleotides (75 base pairs for Operon), printed onto a glass array in a regular arrangement, so that all arrays have an identical oligonucleotide layout [5]. In contrast to Affymetrix and Illumina arrays which measure the transcriptome of one sample at a time, Operon and many other custom arrays utilise two dyes to assess comparative gene expression of two samples on the same chip.

The evaluation of the reproducibility of biological results across microarray platforms is essential for interpreting data independently generated with different technologies and reducing the need to duplicate experiments. Transcriptomic platforms produce both random and systematic errors in predicting actual biological changes. When data from two platforms are compared, the two platform-specific systematic errors are combined with the random error, hence the superior platform which minimises its own systematic errors should have higher agreement with the other platforms.

Growing interest in testing consistency of gene transcription results from different sources has paralleled the increasing number of genome-wide gene expression datasets and the emergence of various microarray platforms and technologies [6]. Some studies comparing data from oligonucleotide arrays and cDNA arrays showed good correlation between intensities and/or fold changes of gene expression [6-8] whilst others reported poor consistency [9,10]. Affymetrix data showed good concordance with those from other oligonucleotide arrays, including Illumina, [11-14] although some studies recommended the use of solely highly expressed genes [8,15].

Studies on multiple platforms have found generally poor reproducibility across gene expression platforms [16,17], although this reproducibility was found to vary between laboratories [18]. The MicroArray Quality Control project, however, compared data from six commercial platforms and an array spotted with Operon oligonucleotides found high inter-platform concordance for differentially expressed genes, as well as better intrasite reproducibility for Illumina and higher intersite concordance for Affymetrix, and fold change reduction for the Operon dataset [6]. Additionally, Quantitative Real Time Polymerase Chain Reaction (qRT-PCR) has been used by a number of studies [17,19-21] as an independent highly reliable gene expression measurement for comparison.

Although numerous methods have been developed for transcriptomic data analysis, the vast majority of these studies used only one method of data normalisation for each platform, and a single agreement measure (for example fold change), hence many methodologically biases may not be recognised. Here we report the first comparative analyses of multiple gene transcription data sets obtained in parallel with different microarray technologies over several popular normalisation methods using multiple cross-platform concordance criteria. We use biologically-relevant transcriptomic data for the mouse and rat genomes in the context of insulin resistance in inbred strains. Probe alignment was performed both by sequence identity and assignment to the Ensembl Gene database. After applying multiple background, intra- and inter-array effect corrections and signal extraction and probe summarisation techniques, we demonstrate the factors affecting the concordance of both magnitude and statistical significance of the transcriptomic effects between platforms.

## Results

To test the performance of widely used microarray technologies in generating consistent genome-wide gene expression data and to assess the effects of various calibration methods on the degree of cross-platform data reproducibility in terms of magnitude and statistical significance of gene expression changes, we used transcriptomic profiles of various mouse and rat organs derived with different microarray systems.

### Analysis of overall levels of inter-platform gene concordance

To determine the degree of consistency in gene content between platforms, we initially tested both individual oligonucleotide mapping and gene level agreements. In the mouse arrays, we aligned 8,901 Illumina oligonucleotides to Affymetrix probe sets using "target sequence" identity and a total of 14,242 Ensembl genes were overlapping between the two platforms. In the rat arrays, analysis of gene level matches via the Ensembl gene database allowed the identification of a core of 1,804 individual genes represented on all three platforms, as opposed to 149 using "target" sequence identity, thus enabling a more reliable three-way comparison to be made.

### Correlation in raw intensity

To test the capacity of the microarray platforms to capture and record changes in probe binding, we then carried out pairwise comparative analysis of the raw intensity signals generated by the platforms. In both rat and mouse datasets, the Pearson correlation (*r*) between the measured intensities for gene matches between any two platforms for all experiments was greater than 0.7, and the majority greater than 0.8. In the rat datasets, the minimal Illumina-Operon pairwise correlations were all greater than 0.8 for all strain comparisons (Table 1). Data from Affymetrix and Illumina arrays were also highly concordant with minimal and maximal correlation values ranging between 0.795 and 0.889, respectively. These high correlation levels imply that the programs used have successfully aligned genes between platforms and that in the vast majority of cases expression of the same genes are measured with different platforms and technologies. However, these results also indicate that platform differences do exist (e.g. hybridisation efficiencies, probe design and data processing), disallowing direct data integration. We therefore analysed changes in gene expression between animals under different experimental conditions as the means of comparison.

**Table 1:** Maximum and minimum intensity correlations between platforms.

		Affymetrix/Illumina		Illumina/Operon		Operon/Affymetrix	
		Max.	Min.	Max.	Min.	Max.	Min.
Liver	BN vs WKY	0.889	0.808	0.848	0.826	0.844	0.776
	GK vs BN	0.887	0.802	0.846	0.824	0.844	0.773
	GK vs WKY	0.887	0.805	0.846	0.824	0.848	0.781
	STZ vs GK	0.887	0.804	0.844	0.823	0.841	0.768
	STZ vs WKY	0.889	0.809	0.845	0.825	0.840	0.772
Kidney	BN vs WKY	0.874	0.795	0.832	0.809	0.828	0.754
	GK vs BN	0.876	0.796	0.827	0.805	0.822	0.749
	GK vs WKY	0.874	0.795	0.829	0.806	0.825	0.751

Data are given for all pairwise platform comparisons for all Affymetrix, Illumina and Operon Ensembl Gene amongst all normalisations, for all liver and kidney rat data.

We verified that genes whose expression level is low compared to all genes on the same microarray produce less reproducible results. This may be caused by the increased influence of background noise on the intensity of the signal generated by the hybridization of the experimental probe to the oligonucleotides. Therefore for many comparisons, all data were filtered so that only the most intense 25% of all oligonucleotides on an array was considered, a comparable proportion to that which the microarray manufacturers' own software calculated for percentage of genes "present" (Additional files 1 and 2).

### Numerical analysis of fold change of gene expression

The magnitude of gene expression changes, alongwith the statistical significance of the effects, is an important criterion in data analysis that assists the selection of genes for further functional investigations. When comparing data across platforms, the signal extraction or calibration techniques used had little impact on the correlations in the logarithm of the absolute intensity measure (Pearson correlations r were approximately 0.8), but had a large impact when correlating fold changes for the Illumina and Affymetrix mouse datasets (see Additional file 3).

In the mouse C57BL/6J diet comparisons, the Affymetrix-Illumina inter-platform correlations in the gene expression fold changes on the logarithmic scale attained using selected normalisations at decreasing levels of filtering for alignments based on sequence identity showed concordant levels with Ensembl gene based alignments and "target matches" (Tables 2 and 3). The correlation was excellent, exceeding 0.7 for all probes for certain pairs of normalisations, approaching 0.9 for the suggested filtering, and surpassing 0.95 when only the most intense signals were analysed. Generally higher inter-platform agreement was found when using the Ensembl Gene alignments.

**Table 2:** Correlations (log2 fold change) between Illumina and Affymetrix mouse "target matches" for selected normalisations.

		Affymetrix normalisations						
Filter (app. N)	Illumina normalisations	Scale – Avgdiff	Quantile-median polish	MAS 5.0	Li-Wong	RMA	GC-RMA	vsn
5% (560)	Quantile	0.936	0.946	0.936	0.941	0.949	0.949	0.946
	Loess	0.940	0.945	0.944	0.944	0.949	0.949	0.954
	Rank	0.941	0.947	0.944	0.945	0.951	0.951	0.953
	vsn	0.940	0.947	0.945	0.947	0.952	0.952	0.953
10% (1125)	Quantile	0.922	0.936	0.920	0.932	0.940	0.944	0.939
	Loess	0.922	0.932	0.925	0.933	0.939	0.943	0.944
	Rank	0.924	0.935	0.925	0.934	0.941	0.944	0.944
	vsn	0.921	0.933	0.926	0.937	0.943	0.946	0.942
25% (2700)	Quantile	0.843	0.875	0.836	0.857	0.893	0.887	0.883
	Loess	0.834	0.868	0.834	0.854	0.888	0.884	0.882
	Rank	0.843	0.874	0.838	0.859	0.894	0.887	0.886
	vsn	0.826	0.860	0.844	0.863	0.897	0.890	0.873
50% (4600)	Quantile	0.760	0.807	0.596	0.730	0.824	0.825	0.816
	Loess	0.746	0.800	0.596	0.725	0.818	0.823	0.813
	Rank	0.759	0.805	0.598	0.731	0.825	0.825	0.817
	vsn	0.724	0.766	0.607	0.728	0.807	0.814	0.780
100% (10018)	Quantile	0.666	0.732	0.289	0.428	0.732	0.739	0.735
	Loess	0.645	0.722	0.285	0.421	0.724	0.735	0.730
	Rank	0.653	0.721	0.286	0.422	0.723	0.731	0.727
	vsn	0.590	0.647	0.280	0.402	0.670	0.674	0.656

The percentages indicate the percentile cut-off for the intensity on both platforms as well as an approximate number of matches (app. N) selected (normalisation-dependent).

**Table 3:** Correlation (log2 fold change) between Illumina and Affymetrix mouse Ensembl gene matches for selected normalisations.

		Affymetrix normalisations						
Filter (app. N)	Illumina normalisations	Scale – Avgdiff	Quantile-median polish	MAS 5.0	Li-Wong	RMA	GC-RMA	vsn
5% (400)	Quantile	0.942	0.949	0.937	0.944	0.951	0.945	0.953
	Loess	0.948	0.950	0.946	0.948	0.952	0.948	0.959
	Rank	0.947	0.951	0.944	0.947	0.953	0.948	0.959
	vsn	0.949	0.954	0.947	0.948	0.957	0.951	0.961
10% (900)	Quantile	0.928	0.938	0.922	0.935	0.947	0.947	0.942
	Loess	0.930	0.936	0.928	0.937	0.946	0.947	0.947
	Rank	0.930	0.937	0.927	0.937	0.947	0.948	0.946
	vsn	0.928	0.935	0.927	0.935	0.946	0.946	0.944
25% (2600)	Quantile	0.864	0.885	0.867	0.869	0.898	0.896	0.894
	Loess	0.860	0.881	0.869	0.866	0.895	0.894	0.895
	Rank	0.865	0.884	0.870	0.869	0.898	0.897	0.896
	vsn	0.858	0.880	0.876	0.874	0.905	0.904	0.892
50% (15600)	Quantile	0.781	0.820	0.689	0.766	0.826	0.825	0.829
	Loess	0.772	0.815	0.688	0.760	0.821	0.822	0.828
	Rank	0.781	0.819	0.691	0.766	0.826	0.825	0.830
	vsn	0.758	0.796	0.695	0.768	0.822	0.819	0.809
100% (14242)	Quantile	0.662	0.724	0.284	0.417	0.712	0.722	0.727
	Loess	0.646	0.718	0.280	0.407	0.704	0.718	0.723
	Rank	0.650	0.715	0.280	0.410	0.704	0.714	0.720
	vsn	0.595	0.652	0.275	0.397	0.660	0.666	0.659

The percentages indicate the percentile cut-off for the intensity on both platforms as well as an approximate number of matches (app. N) selected. This number is normalisation-dependent.

**Figure 1:**
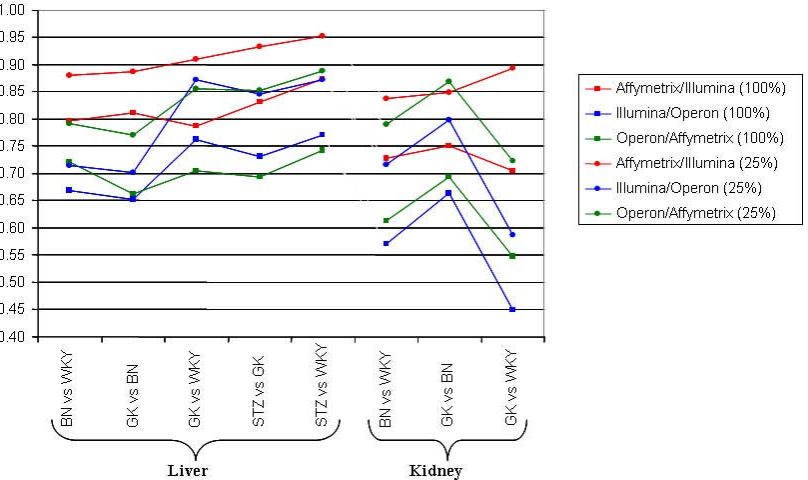
**Comparisons of gene expression changes between platforms in all rat microarray datasets**. Correlation in log2 fold change between Affymetrix, Illumina and Operon Ensembl Gene matches were calculated for all tissues and strain comparisons for all matches (squares) and 25% intensity-filtered data (circles). Illumina data were normalised by loess normalisation for liver and quantile normalisation for kidney. Affymetrix data were normalised by RMA and Operon data by vsn-scale.

For the comparison between gene expression data from rat models derived by Affymetrix, Illumina and Operon microarrays, pairwise platform fold-change correlations, using signal extractions and normalisations selected to maximise the concordance, were derived for all genes and the selection of 25% of genes giving the most intense signals (Figure 1). The liver data set provided higher inter-platform agreements than the kidney results, perhaps due to higher tissue heterogeneity in total kidney than in liver. Multiple strain comparisons were used and often had large impacts on the agreement of the platforms. For the most consistent rat group comparisons (eg. STZvsWKY and GKvsWKY), the correlation between pairs of platforms for intensity-filtered data always exceeded 0.8, with gene level correlation above 0.96 for the STZvsWKY Affymetrix and Illumina comparison. Results from these two platforms were the most concordant, and, if the recommended intensity-based filtering is applied, that the Operon platform agrees most with the Affymetrix platform, suggesting that Affymetrix may be platform generating the most reproducible data.

**Figure 2:**
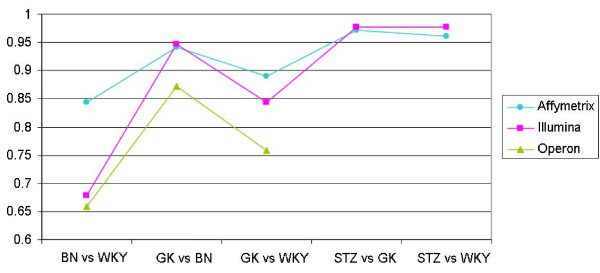
**Correlations between qRT-PCR and microarray results**. Maximum correlations (log2 fold change) between qRT-PCR and array results in rat kidneys are given for all 17 genes in all strain comparisons, maximising correlation over all normalisations.

To verify this, we conducted quantitative RT-PCR (qRT-PCR) analysis of the expression of a selection of genes in the rat kidney (see Additional file 4) and calculated correlations between fold changes of gene expression between the rat models given by qRT-PCR and the three microarray platforms (Figure 2). The correlations between the fold changes generated, and those found by Affymetrix and Illumina were both outstanding, exceeding a Pearson correlation of 0.976. However, these high correlation values may be, at least partly, due to the small number of genes tested, with an over-representation of differentially-expressed genes. Correlation levels with data from the Operon platform were lower for the three comparisons tested (BNvsWKY, GKvsBN and GKvsWKY), but Operon data were not available for the strain comparisons producing the highest correlations between qRT-PCR and Illumina or Affymetrix data (STZvsGK and STZvsWKY).

### Concordance of gene lists

To determine the level of agreement in the list of genes found differentially expressed with the microarray platforms tested, we analysed both fold changes and p values for genes showing the most significantly altered expression. We found that the cross-platform agreement in p values was lower than that of fold changes, which is iin agreement with previous observations [6]. This can be explained by the dependence of p value on variance which is ignored in the fold change calculation and is likely to be platform-dependent.

For the mouse dataset (see Additional files 3, 5, 6), less than half of the matches are in common in Illumina and Affymetrix arrays, which may be lower than expected. For the rat data set, the level of agreement between platforms in gene expression fold changes was dependent on the strain comparison (see Additional file 7). This may due to the important magnitude of the expression differences between the most severely affected rats (STZ-WKY) compared to a control strain (WKY), as the most differentially expressed genes have highly dispersed fold changes, hence easier to reproduce. On the other hand, only small phenotypic differences between two control strains (BN and WKY) may generate much more reduced gene expression differences that may be more susceptible to be masked by variations in the background (see Additional files 1 and 2).

**Figure 3:**
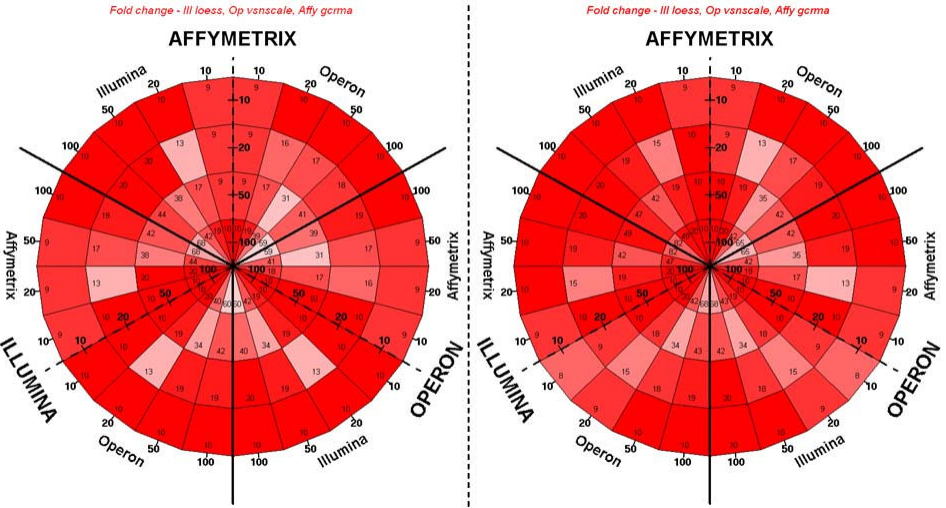
**Cross-platform comparisons of fold changes in the liver transcriptome analyses between WKY-STZ and WKY**. "Dartboard" plots were derived using all Ensembl gene matches between Affymetrix (scale-median polish), Operon (vsn-scale) and Illumina (loess). The number represents the agreement in absolute log2 fold changes for all genes (left, 1,804 genes) and the top 25% most intense genes (right, approximately 280 genes) on all three platforms (see methods). Gene lists generated by the platforms indicated in large block capitals are entered inwards from the circumference and compared to gene lists generated by the other two platforms on either side of the dotted line. Segments are colour coded so that red marks the maximum possible value for a given comparison, and white represents less than half of the maximum.

**Figure 4:**
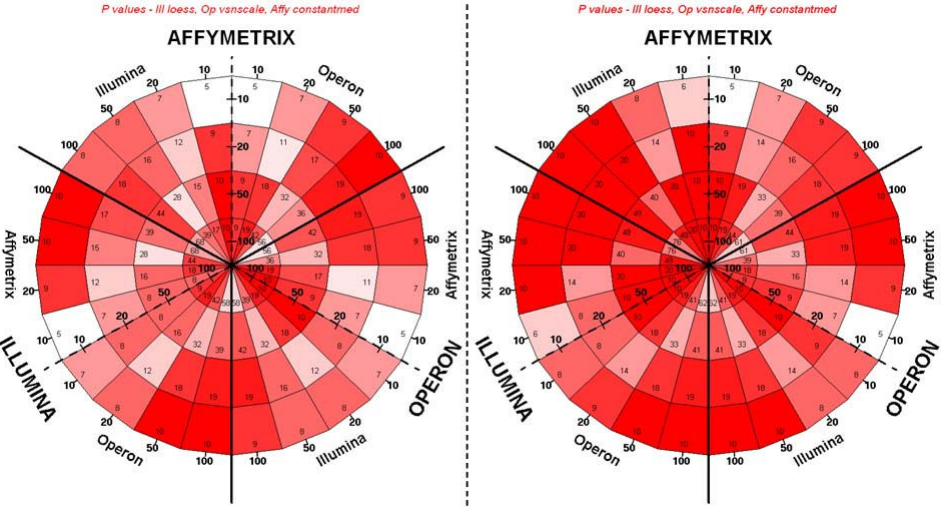
**Cross-platform comparisons of p-values in the liver transcriptome analyses between WKY-STZ and WKY**. "Dartboard" plots were derived using all Ensembl Gene matches between Affymetrix (scale-median polish), Operon (vsn-scale) and Illumina (loess). The number represents the agreement in p values for all genes (left, 1502 genes) and the top 25% most intense genes (right, approximately 280 genes) on all three platforms (see methods). Gene lists generated by the platforms indicated in large block capitals are entered inwards from the circumference and compared to gene lists generated by the other two platforms on either side of the dotted line. Segments are colour coded so that red marks the maximum possible value for a given comparison, and white represents less than half of the maximum.

In order to illustrate the extent of all intra-platform concordances simultaneously, we developed a novel "dartboard" plot (Figures 3 and 4). This system allows the visualisation of strongly concordant or discordant results for subsets of genes selected for various expression fold changes and p values. In the example of gene expression fold changes shown in Figure 3, 17 of the top 20 genes in the Illumina gene list appeared in the top 50 genes as ranked by the Illumina data, whereas all of the top 20 genes from Affymetrix are ranked in the top 50 genes by Illumina. There was excellent agreement in the top fold change lists of genes differentially expressed for all three platforms (Figure 3 and Additional file 7), again greater than for the p values (Figure 4 and Additional file 8). These results indicate that if the highly-differentially expressed genes are present on all arrays, they will reliably be called by any of these platforms. However, in this experiment the number of genes on each platform was very different, so that the full lists will be more dissimilar. Overall, Affymetrix achieved high correlations over all strain comparisons, whereas Illumina ranked highest for the comparisons with the strongest transcriptomic divergences, although this difference is marginal. This suggests that Illumina may be the most appropriate platform when investigating highly expressed genes with large fold changes and Affymetrix more sensitive when investigating marginal gene expression changes.

#### Effects of normalisation methods on inter-platform concordance

##### Numerical analysis of fold change of gene expression

Gene expression profiling data were used to compare the various calibration techniques employed in this study. The signal extraction model had a much larger effect on the analysis than the subsequent normalisation (data not shown). The Affymetrix signal extractions showed a much larger heterogeneity than the background corrections and normalisations used on the other platforms. This is largely due to the more complex design of the Affymetrix microarrays, including the presence of distinct oligonucleotides in a probe set and mismatch probes, which are handled differently. The difference was greatest at low fluorescence. In the mouse dataset, the Li-Wong method and MAS 5.0 had high variance (Tables 2 and 3) and filtering was required. In terms of correlation in fold change of gene expression, RMA, GC-RMA and vsn consistently showed the highest agreement with other platforms, whereas MAS 5.0 and the Li-Wong method require high levels of filtering for a similar performance. For Illumina, the differences were less marked with the vsn method producing slightly lower inter-platform correlations. The Operon methods showed little discordance.

When qRT-PCR data derived in rats for a small election of genes were used to assess the effects of normalisation methods on microarray-based gene expression data, we found that for Affymetrix, MAS 5.0, RMA, GC-RMA and the Li-Wong method all had very high correlations with the logarithm of the qRT-PCR fold change, and the highest was comparison-dependent (see Additional file 9). For Illumina, vsn normalisation correlated the most with the qRT-PCR data, although only small differences were observed (see Additional file 10). For Operon (see Additional file 11), the vsn transformation correlates least of all, with the Kooperberg background correction producing the most concordance.

In the mouse data set, the Affymetrix methods without background correction showed similar magnitude of fold changes to all techniques applied to Illumina data, except vsn, whereas the other Affymetrix methods showed higher fold changes (see Additional file 12). For the rat data set, we are able to analyse for each rat group comparison, fold changes obtained for each platform against those generated by qRT-PCR (see Additional file 13). All Operon techniques slightly over-estimated the fold change, whereas all Illumina data slightly under-estimated the change compared to qRT-PCR. For Affymetrix, MAS 5.0 and GC-RMA were most accurate, RMA and Li-Wong provided slight under-estimates, and the use of PM values only led to a large under-estimation of fold changes.

##### Concordance in gene lists

Finally we tested the effect of normalisation methods on the concordance in the lists of genes found differentially expressed with different platforms. Amongst Affymetrix signal extraction methods, MAS 5.0 showed the lowest agreement in fold change and p value when using all matches, but agreement was good with high filtering (Tables 2 and 3, Additional file 12). The Li-Wong method provided low inter-platform fold change correlation, but excellent agreements in p values and (for only the most extreme filtered oligonucleotides) fold changes. The RMA and GC-RMA performed well in these tests. No consistent differences were found for Illumina normalisations. The loess normalisation was superior for the Illumina experiment in rat liver, but it was quantile normalisation which agreed most for the rat kidney transcriptomic dataset. For Operon data, using the vsn transformation had the highest agreements in the top gene lists sorted by absolute fold change or p value.

Overall, when no filtering was applied, the factors determining most concordance between platforms, in approximate order of importance, were: Affymetrix signal extraction, group comparison, organ and between-array normalisations (all platforms) (see for example Additional files 7 and 8). If filtering was applied, however, group comparisons and organ were most important with more similar performance by all signal extractions.

## Discussion

We have carried out an extensive assessment of the performance of three gene expression profiling platforms designed for two mammalian genomes and provide information on analytical methods that are best suited to processing data from specific types of arrays. Even though comparisons of data from multiple microarray technologies have been extensively tested [7,21], including in advanced high density array systems such as Illumina and Affymetrix [11], we more specifically focused on the impact of normalisation methods on multiple concordance criteria (raw intensity, expression ratio, statistical significance), which remain partly addressed in the literature, to assess the extent of cross-platform data consistency and divergence. Overall, using genome-wide gene expression profiling data of rat and mouse genomes we provide confirmatory evidence of the extremely high agreement between platforms as previously suggested [8], and the particularly high consistency of results at a gene level between Illumina and Affymetrix [11]. Both platforms agree less well with Operon-generated data, and this technology also correlates less well with independent qRT-PCR data. Both Illumina and Affymetrix had outstanding correlation with qRT-PCR results, indicating that they produce highly reliable fold change results on a gene level, even for a modest number of biological replicates.

Multiple strain comparisons were used and often had large impacts on the agreement of results from the platforms, due mainly to genetic differences. The comparison between inbred rat strains for hepatic and renal gene expression, which was repeated on Affymetrix, Illumina and Operon microarrays, showed that the kidney appeared to produce less reproducible data, perhaps due to morphological heterogeneity of this organ. However, the correlation between the fold changes generated by qRT-PCR, a highly reliable method of measuring transcription [20,22] and those found by Affymetrix and Illumina were outstanding, exceeding a Pearson correlation of 0.976.

Perhaps surprisingly, correlations achieved between these microarray systems and the independent qRT-PCR technique were higher than the inter-platform comparisons using sequence or Ensembl Gene matching techniques, and even higher than between distinct normalisations on the same platform. However, those methods attempted to match oligonucleotides on a genome-wide level, while qRT-PCR comparisons use a small number of known genes, specifically chosen for their biological role and/or high differential expression in the microarray experiments.

Data from our cross-platform comparisons were improved by methods for probe alignment, which were based on sequence identity and assignment to the Ensembl. The majority of published cross-platform analyses have used methods based on identifiers (eg. gene names and accession numbers), which represent significant challenges due to the existence of synonyms and evolving or inconsistent annotations. Our inter-platform correlations in log fold changes and agreements in the most affected genes obtained using "target" sequence identity were often surpassed by aligning the oligonucleotides to Ensembl gene sequences. This is surprising, as the precise matching of oligonucleotide sequences within a gene provided by "target matching" is expected to be most reproducible, as the effects of complicating factors, such as alternative splicing, are reduced. However, the removal of poorly annotated probes in a probe set and combining information across entire genes create more reproducible results, and demonstrate the power of this novel alignment tool.

The Affymetrix signal extractions showed a much larger heterogeneity than the background corrections and normalisations used on the other platforms. This is largely due to the way the methods treat the more complicated design of the Affymetrix platform (distinct probe sequences in a probe set and MM probes).

RMA was the method which performed consistently well in all comparisons. It produced high correlation in log fold change of gene expression between platforms, regardless of filtering, annotation or biological comparison and high agreement in gene lists for both statistical significance and fold change magnitude. However, the fold change can be underestimated by RMA [23]. This was suggested by the mouse study, in comparison with Illumina, and the rat kidney comparison with qRT-PCR data, although fold changes of gene expression in the rat datasets were comparable with those from Illumina and Operon arrays. The related method GC-RMA also performed very well in a large number of comparisons [24], correlating the most in terms of intensity, having high correlation in gene expression fold changes, high agreement in most gene lists, and producing comparable gene expression fold changes with qRT-PCR. However, it produced lower correlations with Operon and lower p value agreement for the rat liver dataset.

The Affymetrix own method MAS 5.0 and the popular Li-Wong method showed very mixed results, especially extreme for MAS 5.0. These methods showed poor agreement with other platforms when all genes were used, but occasionally produced excellent results when intensity-based filtering was utilised. These methods often showed extremely high agreement in top fold change and (especially Li-Wong) p value lists. These two methods also produced very high correlations with the qRT-PCR data, and MAS 5.0 fold change was almost identical. These findings suggest that these methods may be useful when searching for large effect sizes in highly-expressed known genes, but are poor for whole genome studies or detection of effects of small magnitude. Other Affymetrix normalisations, which used the PM values without correction often showed intermediate results. This implies that using either the mismatch values or a statistical framework to calculate and remove background effects is likely to improve the accuracy, sensitivity and reproducibility of transcriptomic data generated using the Affymetrix platform.

## Conclusion

Testing multiple analytical methods in microarray experiments is essential to maximise the robustness of results of gene transcription profiling in terms of metabolic and hormonal regulations of systems and interpretations of biological processes. This study provides an exhaustive and stringent assessment of analytical methods best suited for processing data from different microarray-based gene expression profiling technologies. These results are important for comparing transcriptomic data generated with different platforms and explaining inconsistencies between results that can be reanalysed using the most appropriate method. Our analyses can assist the integration of gene expression data obtained with different technologies in a single biological system.

Based on results from our analyses, the following recommendations can be made:

1. The choice of platform depends on the design of the experiment. Illumina microarrays can be more appropriate for models with large transcriptomic differences, whereas Affymetrix have a larger dynamic range.

2. For Affymetrix studies, the choice of signal extraction significantly affects reproducibility. RMA performs well in all tests, whereas MAS 5.0 and the Li-Wong method have high accuracy and sensitivity in detecting highly differentially expressed genes.

3. Filtering results on intensity level, through platform-specific present calls, is very important for obtaining reliable data.

4. In agreement with published findings [6], for moderate sample sizes, comparing gene lists through p values leads to an observed low concordance of results between datasets, due to the reliance of the *t*-test statistic on the unstable estimate of sample error, and the comparison of observed fold changes is recommended.

5. Comparing platforms on a gene-level summary basis rather than individual nucleotide or accession number improves data reproducibility for the arrays studied.

## Methods

### Animals and RNA sample preparation

Male mice of the inbred strain C57BL/6JOxjr were bred locally using stocks from the Jackson laboratory. They are referred as C57BL/6 throughout the text. Mice were fed a normal carbohydrate (CHD) diet (S&K Universal Ltd, Hull, UK) or a 40% high fat diet (HFD) (Special Diet Services, Witham, UK) *ad libitum *as previously described [25]. For the rat data set, we used biopsies of male inbred rats of the diabetic Goto-Kakizaki (GK) strain [26], the normoglycaemic Brown-Norway (BN) and Wistar-Kyoto (WKY, also referred as W in this study) strains, and WKY rats made diabetic (approximately 16 mM) by intravenous injection of a solution of Streptozotocin (Sigma-Aldrich, Poole, UK) at 75 mg/kg in citrate buffer (WKY-STZ rats also referred as STZ throughout the article). Rats of the GK and BN strains were from the Oxford colony (GK/Ox, BN/Ox) and WKY rats were purchased from a commercial supplier (Harlan, UK). All animals were kept under identical standard maintenance conditions on 12 h light/dark cycle. All experiments were carried out in accordance with national guidelines.

Organs used for gene expression profiling were chosen for their role in glucose homeostasis (liver) and diabetic complications (kidney). Total RNA was prepared from liver (mice and rats) and kidney (rat) biopsies as previously described [25].

### Microarray hybridisation

Targets prepared from C57/Bl6 mice (n = 5 per diet group) were individually hybridised to both Affymetrix^® ^GeneChip^® ^Mouse Genome 430 2.0 arrays, which contain 45,101 probe sets, and Illumina^® ^Sentrix^® ^BeadChip Mouse-6 Expression arrays (Beta Version 1), which contain 46,120 distinct oligonucleotide sequences.

Three separate types of microarray were used to carry out rat gene expression profiling:

- Operon Rat v1.0 OpArray™ is a two-colour gene expression system, containing 5,717 oligonucleotides, almost entirely representative for unique well-documented genes. A full factorial dye-swap design was implemented, where a target from each animal's mRNA was hybridised on a separate array with all animals from different strains, and each array was repeated with the corresponding dyes switched, to correct for dye biases. A total of 108 slides were used for the liver experiment and 54 for the kidney, as three animals per group were used. RNA from WKY-STZ kidneys were not used on this array type.

- Affymetrix^® ^GeneChip^® ^Rat Expression Set 230A GeneChip^® ^arrays contain 15,923 probe sets, corresponding to over 10,000 distinct annotated genes. A total of twelve microarrays were hybridised using samples from three animals for each of the four groups.

- Beta test version of the Illumina^® ^Sentrix^® ^BeadChip RatRef-12_V1_Eval Expression microarrays carry twelve arrays for every slide, and each array contained 22,612 distinct oligonucleotides. Technical replicates were used for all samples.

All experiments are MIAME compliant. Protocols and data are available through ArrayExpress  under the accession E-MEXP-1195 (Rat kidney transcriptome on Affymetrix), E-MEXP-889 (Rat liver transcriptome on Affymetrix), E-TABM-500 (Rat kidney transcriptome on Operon), E-TABM-502 (Rat kidney transcriptome on Illumina), E-MEXP-1755 (C57BL/6J mouse liver transcriptome on Affymetrix).

### Background removal and signal extraction techniques

These techniques are very platform-dependent because of the various background measurement techniques and scanning technologies. Different signal extraction techniques, especially for the Affymetrix platform, are expected to be less comparable than different between-array normalisations applied to the same signal extraction, as the extraction models glean distinct information from the raw foreground (and background) intensities, whereas the normalisation methods seek to minimise distribution differences through global corrections. For Operon arrays we used background subtraction, Normal and Exponential Convolution model (normexp) [27], and methods proposed by Kooperberg et al. [28] and Edwards [29]. For Affymetrix arrays, we applied the microarray Suite version 5.0 (MAS 5.0) [30], the model-based expression indexes (MBEI) [31,32] (Li-Wong method), the Robust Multi-Array Analysis (RMA) [23], the GC-RMA method [24], using the raw Perfect Match values with no background corrections [33]. Finally, the Illumina platform does not directly measure any background or non-specific hybridisation control. However, this platform has been shown to have high precision (Kuhn *et al. *2004) and is analysed, by default, with no background removal technique.

The variance stabilisation (vsn) method [34] was used for both within- and between-array calibration, and was used for data generated by all three platforms. We used scale transformation [35], quantile normalisation [36], local regression (loess) [35], including print-tip loess normalisation for Operon [35] and cubic spline fitting [37]. All calculations used were either conducted in the R Language and Environment for Statistical Computing (R) [38] or the Illumina^® ^BeadStudio^® ^software. All the R normalisations were implemented in the "LIMMA" [27], "affy" [39] or "vsn" [34] R packages.

### Data validation by quantitative RT-PCR

Quantitative Real-Time Polymerase Chain Reaction (qRT-PCR) was performed for a total of 17 genes with kidney samples from all four rat models. Genes were selected primarily due to differential expression in either the Affymetrix or Operon-generated rat kidney microarray data sets. Experiments were conducted using samples from the same animals as profiled with microarrays, and technical triplicates were used for all genes. Actin was used as the control "house-keeping" gene.

### Gene annotations in the array systems

We labelled as cross-platform "target matches", sets where the oligonucleotide sequence of a probe aligned identically to the sequence spanned by all probes of an Affymetrix probe set. Dai et al. observed that Affymetrix probe set design for each microarray could not evolve as the genome and transcriptome information improved over time, and that, in some cases, probes in the same probe set derived from distinct genes [40]. They created new annotation files based on public annotations, and claim 30–50% discrepancy in genes previously identified as differentially-expressed. Due to the redundancy in most other databases, alignments to Ensembl genes were used. The new probe sets are formed by identifying all perfect match (PM) probes which have a unique identical match amongst Ensembl genes, and forcing the new probe sets to contain at least three probes.

Publicly available gene annotations were used . For Illumina and Operon, no such annotations existed in the literature. However, as on both these platforms oligonucleotides in a set are identical, we aligned all probes against Ensembl genes , with the version used for the Affymetrix probe sets, to ensure the same genome builds were consistently used (Mouse NCBI m34 and Rat RGSC 3.4).

### Statistical issues

Prior to analysis, the data was filtered by removing a user-specified proportion of oligonucleotides with the lowest mean log2 intensity in the comparison of interest. Only matches where at least one oligonucleotide from each platform under investigation remained after filtering were used.

As different signal extractions and normalisations produce outputs in absolute or logarithmic values (in varying bases), all expression intensities and fold changes from all platforms were converted into logarithmic (base 2) values after normalisation. This transformation converts multiplicative effects (such as fold changes in gene expression) into additive effects, which increases ease of both analysis and interpretation [41]. Pearson correlations were used throughout this study. The correlation in intensity is less indicative than the correlation in fold change, as different platforms will have differing hybridisation efficiencies that may be sample dependent, or vary more through different normalisation techniques, but the intensity should be proportional to the abundance of mRNA in the sample, so fold changes should be conserved.

We also investigated whether the platforms produce the same genes as most worthy of investigation, if one only considers the "top" genes ranked by either most significant p value or highest absolute fold change. Although any overlap is likely to be very highly pointwise significant when compared to the null hypothesis of no relation between the platforms, whether the agreement is sufficient to be biologically useful is difficult to assess.

When comparing all three platforms, three pairwise tables would have to be created. In order to illustrate all intra-platform concordances simultaneously, a new plot which was labelled a "dartboard plot" was specifically designed (Figures 3 and 4). The platforms in large block capitals have their lists entered radially from the circumference, and apply until the thick black lines, the other platforms are to either side of the dotted line and their lists move out from the dotted line. The segments, corresponding to using all or part of the dataset, are colour-coded so that red marks the maximum possible value for that segment, i.e. the minimum of the two list sizes, and white represents 50% or fewer matches. Note that segments on either side of a thick black line use the same data.

It must be emphasised that when compiling these concordance lists, only oligonucleotides or genes which match to the other platform or platforms are included, implying there are no missing values, so that the above statistics can be applied and the lists directly compared for their technical performance. However, this is an important concern for experimental reasons; if two platforms show high concordance for genes assayed on both, but one measures the expression of many more genes, the two will not be of equivalent biological value. For the mouse experiment, the Affymetrix and Illumina microarrays had very similar oligonucleotide numbers, while in the rat the Operon platform had fewer oligonucleotides and the Affymetrix GeneChip utilised was designed in a prior generation of feature size to the Illumina BeadChip, so fewer probe sets were present. Hence for a valid comparison, only matching oligonucleotides were used. In general, similar generation Affymetrix and Illumina microarrays contain similar oligonucleotide numbers.

## Abbreviations

BN: Brown-Norway; GK: Goto-Kakizaki; LIMMA: Linear Models for Microarray Analysis; MAS5.0: MicroArray Suite version 5.0; RMA: Robust Multi-array Analysis; STZ: Streptozotocin; WKY: Wistar-Kyoto; MM: mismatch; PM: perfect match; qRT-PCR: quantitative real time PCR; vsn: variance stabilisation.

## Authors' contributions

AS and JR produced the Operon arrays. AS, KA, MTB and PJK performed gene transcription profiling experiments and data interpretation. SPW carried out statistical analyses, bioinformatic studies and gene annotations of microarray data. SPW and DG drafted the manuscript. DG, PJK and MTB conceived of the study, and participated in its design and coordination. All authors read and approved the final manuscript.

## Supplementary Material

Additional file 1 **Scatterplots of mouse target log2 fold changes for Illumina (*y*) against Affymetrix (*x*) for most normalisations for the 25% most intense oligonucleotides.** Effects of normalisation methods on gene expression changes derived by Illumina and Affymetrix. Click here for file

Additional file 2 **Scatterplots of all rat Ensembl Gene match log2 fold changes between Affymetrix (normalised by RMA), Illumina (normalised by loess for liver and quantile normalisation for kidney) and Operon (normalised by vsn and quantile normalisation) for all tissues and strain comparisons.** Analysis of rat gene expression changes for genes represented on the Illumina, Affymetrix and Operon arrays. Click here for file

Additional file 3 **Scatterplots of Rat Ensembl Gene match log2 fold changes between Affymetrix (normalised by RMA), Illumina (normalised by loess for liver and quantile normalisation for kidney) and Operon (normalised by vsn and quantile normalisation) for all tissues and strain comparisons, restricting analysis to the 25% most intense genes on each platform (approximately 320 genes).** Analysis of the magnitude of rat gene transcription changes for genes represented on the Illumina, Affymetrix and Operon arrays and showing strong level of expression. Click here for file

Additional file 4 **Gradient of log2 fold change scatterplots of Illumina (*y*) against Affymetrix (*x*) mouse "Target matches" for all normalisations, restricting analysis to the 25% most intense oligonucleotides (approximately 2,700 oligonucleotides).** Effects of various normalisation methods on mouse gene expression changes generated by Illumina and Affymetrix arrays. Click here for file

Additional file 5 **Concordance of all mouse Affymetrix and quantile normalised Illumina unique "Target match" p value data between different Affymetrix normalisations (8,886 matches).** Comparative analysis of statistical significance of mouse gene expression data derived by Illumina and Affymetrix arrays. Click here for file

Additional file 6 **Concordance of all mouse Affymetrix and quantile normalised Illumina unique "Target match" fold change data between different Affymetrix normalisations (8,886 matches).** Comparative analysis of the magnitude of mouse gene expression changes derived by Illumina and Affymetrix arrays. Click here for file

Additional file 7 **Total concordance in top *X *fold change lists between Affymetrix (normalised by GC-RMA), Illumina (normalised by loess for liver data and quantile for kidney data) and Operon (vsn and scale) for both tissues and all comparisons, using unfiltered (1,804 genes) and top 25% intensity-based filtering (approximately 280 genes).** Comparative analysis of the magnitude of rat gene expression changes derived by Illumina, Affymetrix and Operon arrays. Click here for file

Additional file 8 **Total concordance in top *X *p value lists between Affymetrix (normalised by scale and median polish), Illumina (loess) and Operon (vsn and scale) for both tissues and all comparisons, using unfiltered (1,502 genes) and top 25% intensity-based filtering (approximately 280 genes).** Comparative analysis of the statistical significance of rat gene expression changes derived by Illumina, Affymetrix and Operon arrays. Click here for file

Additional file 9 **Correlation in log2 fold change between qRT-PCR and all Affymetrix normalisations by strain comparison for all seventeen genes in rat kidney.** Comparative analysis of gene expression changes given by quantitative RT-PCR and Affymetrix array data normalised using several methods. Click here for file

Additional file 10 **Correlation in log2 fold change between qRT-PCR and all Illumina normalisations by strain comparison for all seventeen genes in rat kidney.** Comparative analysis of gene expression changes given by quantitative RT-PCR and Illumina array data normalised using several methods. Click here for file

Additional file 11 **Correlation in log2 fold change between qRT-PCR and all Operon normalisations by strain comparison for all seventeen genes in rat kidney.** Comparative analysis of gene expression changes given by quantitative RT-PCR and Operon array data normalised using several methods. Click here for file

Additional file 12 **Descriptions and log2 fold changes for all seventeen genes selected for qRT-PCR in all strain comparisons for the rat kidney experiment.** Gene expression ratios derived by quantitative RT-PCR of renal samples from diabetic and control rats. Click here for file

Additional file 13 **Gradient of log2 fold change fit of qRT-PCR (*y*) against microarray (*x*) for seventeen genes for selected normalisations for all microarray platforms for all rat kidney comparisons.** Comparative analysis of renal gene expression ratios in rat models of diabetes and controls given by quantitative RT-PCR and normalised data from the corresponding genes on Illumina, Affymetrix and Operon arrays. Click here for file

## References

[B1] Schena M, Shalon D, Davis RW, Brown PO (1995). Quantitative monitoring of gene expression patterns with a complementary DNA microarray. Science.

[B2] Lockhart DJ, Dong H, Byrne MC, Follettie MT, Gallo MV, Chee MS, Mittmann M, Wang C, Kobayashi M, Horton H (1996). Expression monitoring by hybridization to high-density oligonucleotide arrays. Nat Biotechnol.

[B3] Kuhn K, Baker SC, Chudin E, Lieu MH, Oeser S, Bennett H, Rigault P, Barker D, McDaniel TK, Chee MS (2004). A novel, high-performance random array platform for quantitative gene expression profiling. Genome Res.

[B4] Michael KL, Taylor LC, Schultz SL, Walt DR (1998). Randomly ordered addressable high-density optical sensor arrays. Anal Chem.

[B5] Rat OpArray datasheet Version 3.0. http://www.operon.com/products/microarrays/oparrays_download.aspx.

[B6] Shi LM, Tong WD, Fang H, Scherf U, Han J, Puri RK, Frueh FW, Goodsaid FM, Guo L, Su ZQ (2005). Cross-platform comparability of microarray technology: Intra-platform consistency and appropriate data analysis procedures are essential. BMC Bioinformatics.

[B7] Yauk CL, Berndt ML, Williams A, Douglas GR (2004). Comprehensive comparison of six microarray technologies. Nucleic Acids Res.

[B8] Jarvinen AK, Hautaniemi S, Edgren H, Auvinen P, Saarela J, Kallioniemi OP, Monni O (2004). Are data from different gene expression microarray platforms comparable?. Genomics.

[B9] Kuo WP, Jenssen TK, Butte AJ, Ohno-Machado L, Kohane IS (2002). Analysis of matched mRNA measurements from two different microarray technologies. Bioinformatics.

[B10] Tan PK, Downey TJ, Spitznagel EL, Xu P, Fu D, Dimitrov DS, Lempicki RA, Raaka BM, Cam MC (2003). Evaluation of gene expression measurements from commercial microarray platforms. Nucleic Acids Res.

[B11] Barnes M, Freudenberg J, Thompson S, Aronow B, Pavlidis P (2005). Experimental comparison and cross-validation of the Affymetrix and Illumina gene expression analysis platforms. Nucleic Acids Res.

[B12] Barczak A, Rodriguez MW, Hanspers K, Koth LL, Tai YC, Bolstad BM, Speed TP, Erle DJ (2003). Spotted long oligonucleotide arrays for human gene expression analysis. Genome Research.

[B13] Woo Y, Affourtit J, Daigle S, Viale A, Johnson K, Naggert J, Churchill G (2004). A comparison of cDNA, oligonucleotide, and Affymetrix GeneChip gene expression microarray platforms. J Biomol Tech.

[B14] Petersen D, Chandramouli GVR, Geoghegan J, Hilburn J, Paarlberg J, Kim CH, Munroe D, Gangi L, Han J, Puri R (2005). Three microarray platforms: an analysis of their concordance in profiling gene expression. Bmc Genomics.

[B15] Shippy R, Sendera TJ, Lockner R, Palaniappan C, Kaysser-Kranich T, Watts G, Alsobrook J (2004). Performance evaluation of commercial short-oligonucleotide microarrays and the impact of noise in making cross-platform correlations. Bmc Genomics.

[B16] Bammler T, Beyer RP, Bhattacharya S, Boorman GA, Boyles A, Bradford BU, Bumgarner RE, Bushel PR, Chaturvedi K, Choi D (2005). Standardizing global gene expression analysis between laboratories and across platforms. Nat Methods.

[B17] Kuo WP, Liu F, Trimarchi J, Punzo C, Lombardi M, Sarang J, Whipple ME, Maysuria M, Serikawa K, Lee SY (2006). A sequence-oriented comparison of gene expression measurements across different hybridization-based technologies. Nat Biotechnol.

[B18] Irizarry RA, Warren D, Spencer F, Kim IF, Biswal S, Frank BC, Gabrielson E, Garcia JG, Geoghegan J, Germino G (2005). Multiple-laboratory comparison of microarray platforms. Nat Methods.

[B19] Larkin JE, Frank BC, Gavras H, Sultana R, Quackenbush J (2005). Independence and reproducibility across microarray platforms. Nature Methods.

[B20] Qin LX, Beyer RP, Hudson FN, Linford NJ, Morris DE, Kerr KF (2006). Evaluation of methods for oligonucleotide array data via quantitative real-time PCR. BMC Bioinformatics.

[B21] Shi L, Reid LH, Jones WD, Shippy R, Warrington JA, Baker SC, Collins PJ, de Longueville F, Kawasaki ES, Lee KY (2006). The MicroArray Quality Control (MAQC) project shows inter- and intraplatform reproducibility of gene expression measurements. Nat Biotechnol.

[B22] Verhaak RGW, Staal FJT, Valk PJM, Lowenberg B, Reinders MJT, de Ridder D (2006). The effect of oligonucleotide microarray data pre-processing on the analysis of patient-cohort studies. BMC Bioinformatics.

[B23] Irizarry RA, Bolstad BM, Collin F, Cope LM, Hobbs B, Speed TP (2003). Summaries of Affymetrix GeneChip probe level data. Nucleic Acids Res.

[B24] Wu ZJ, Irizarry RA, Gentleman R, Martinez-Murillo F, Spencer F (2004). A model-based background adjustment for oligonucleotide expression arrays. Journal Of The American Statistical Association.

[B25] Toye AA, Dumas ME, Blancher C, Rothwell AR, Fearnside JF, Wilder SP, Bihoreau MT, Cloarec O, Azzouzi I, Young S (2007). Subtle metabolic and liver gene transcriptional changes underlie diet-induced fatty liver susceptibility in insulin-resistant mice. Diabetologia.

[B26] Goto Y, Kakizaki M, Masaki N (1975). Spontaneous Diabetes produced by selective breeding of normal Wistar Rats. Proc Japan Acad.

[B27] Smyth GK, Gentleman R, Carey VJ, Dudoit S, Irizarry R, Huber W (2005). Limma: linear models for microarray data. Bioinformatics and Computational Biology Solutions using R and Bioconductor.

[B28] Kooperberg C, Fazzio TG, Delrow JJ, Tsukiyama T (2002). Improved background correction for spotted DNA microarrays. J Comput Biol.

[B29] Edwards D (2003). Non-linear normalization and background correction in one-channel cDNA microarray studies. Bioinformatics.

[B30] Liu WM, Mei R, Di X, Ryder TB, Hubbell E, Dee S, Webster TA, Harrington CA, Ho MH, Baid J (2002). Analysis of high density expression microarrays with signed-rank call algorithms. Bioinformatics.

[B31] Li C, Wong WH (2001). Model-based analysis of oligonucleotide arrays: model validation, design issues and standard error application. Genome Biol.

[B32] Li C, Wong WH (2001). Model-based analysis of oligonucleotide arrays: expression index computation and outlier detection. Proc Natl Acad Sci USA.

[B33] Harr B, Schlotterer C (2006). Comparison of algorithms for the analysis of Affymetrix microarray data as evaluated by co-expression of genes in known operons. Nucleic Acids Research.

[B34] Huber W, von Heydebreck A, Sultmann H, Poustka A, Vingron M (2002). Variance stabilization applied to microarray data calibration and to the quantification of differential expression. Bioinformatics.

[B35] Yang YH, Dudoit S, Luu P, Lin DM, Peng V, Ngai J, Speed TP (2002). Normalization for cDNA microarray data: a robust composite method addressing single and multiple slide systematic variation. Nucleic Acids Res.

[B36] Yang YH, Thorne NP (2003). Normalization for two-color cDNA microarray data. Science and Statistics: A Festschrift for Terry Speed, IMS Lecture Notes.

[B37] Workman C, Jensen LJ, Jarmer H, Berka R, Gautier L, Nielser HB, Saxild HH, Nielsen C, Brunak S, Knudsen S (2002). A new non-linear normalization method for reducing variability in DNA microarray experiments. Genome Biol.

[B38] Ihaka R, Gentleman R (1996). R: A Language for Data Analysis and Graphics. J Comput Graph Statist.

[B39] Irizarry R, Gautier L, Bolstad B, C M (2003). affy: Methods for Affymetrix Oligonucleotide Arrays. R package versions 1.1.2–1.10.0.

[B40] Dai M, Wang P, Boyd AD, Kostov G, Athey B, Jones EG, Bunney WE, Myers RM, Speed TP, Akil H (2005). Evolving gene/transcript definitions significantly alter the interpretation of GeneChip data. Nucleic Acids Res.

[B41] Kerr MK, Churchill GA (2001). Experimental design for gene expression microarrays. Biostatistics.

