# Supplementary material for: Comparative analysis of methods for gene transcription profiling data derived from different microarray technologies in rat and mouse models of diabetes
Source: BMC Genomics. 2009 Feb 5;10:63. doi: 10.1186/1471-2164-10-63 (PMC2652496; doi:10.1186/1471-2164-10-63)

Additional file 1. Scatterplots of mouse target log2 fold changes for Illumina (*y*) against Affymetrix (*x*) for most normalisations for the 25% most intense oligonucleotides.

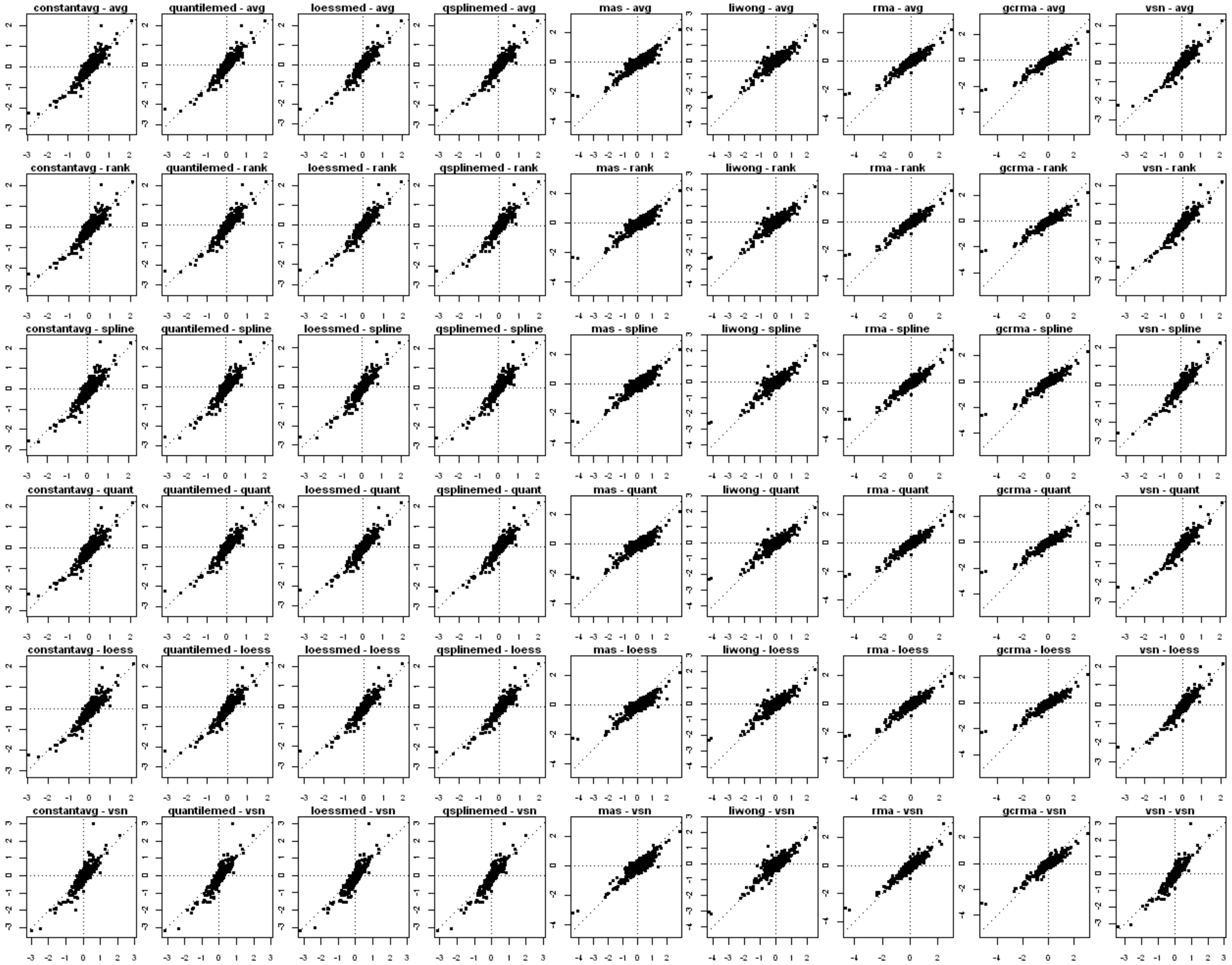

Supplement: Additional file 1 — Scatterplots of mouse target log2 fold changes for Illumina (y) against Affymetrix (x) for most normalisations for the 25% most intense oligonucleotides. Effects of normalisation methods on gene expression changes derived by Illumina and Affymetrix. [file 1471-2164-10-63-S1.pdf]
