# Supplementary material for: Comparative analysis of methods for gene transcription profiling data derived from different microarray technologies in rat and mouse models of diabetes
Source: BMC Genomics. 2009 Feb 5;10:63. doi: 10.1186/1471-2164-10-63 (PMC2652496; doi:10.1186/1471-2164-10-63)

Additional file 2. Scatterplots of all rat Ensembl Gene match log2 fold changes between Affymetrix (normalised by RMA), Illumina (normalised by loess for liver and quantile normalisation for kidney) and Operon (normalised by vsn and quantile normalisation) for all tissues and strain comparisons. The top row plots Illumina (*y*-axis) against Affymetrix (*x*-axis), the second row plots Operon (*y*-axis) against Illumina (*x*-axis) and the third row plots Affymetrix (*y*-axis) against Operon (*x*-axis).

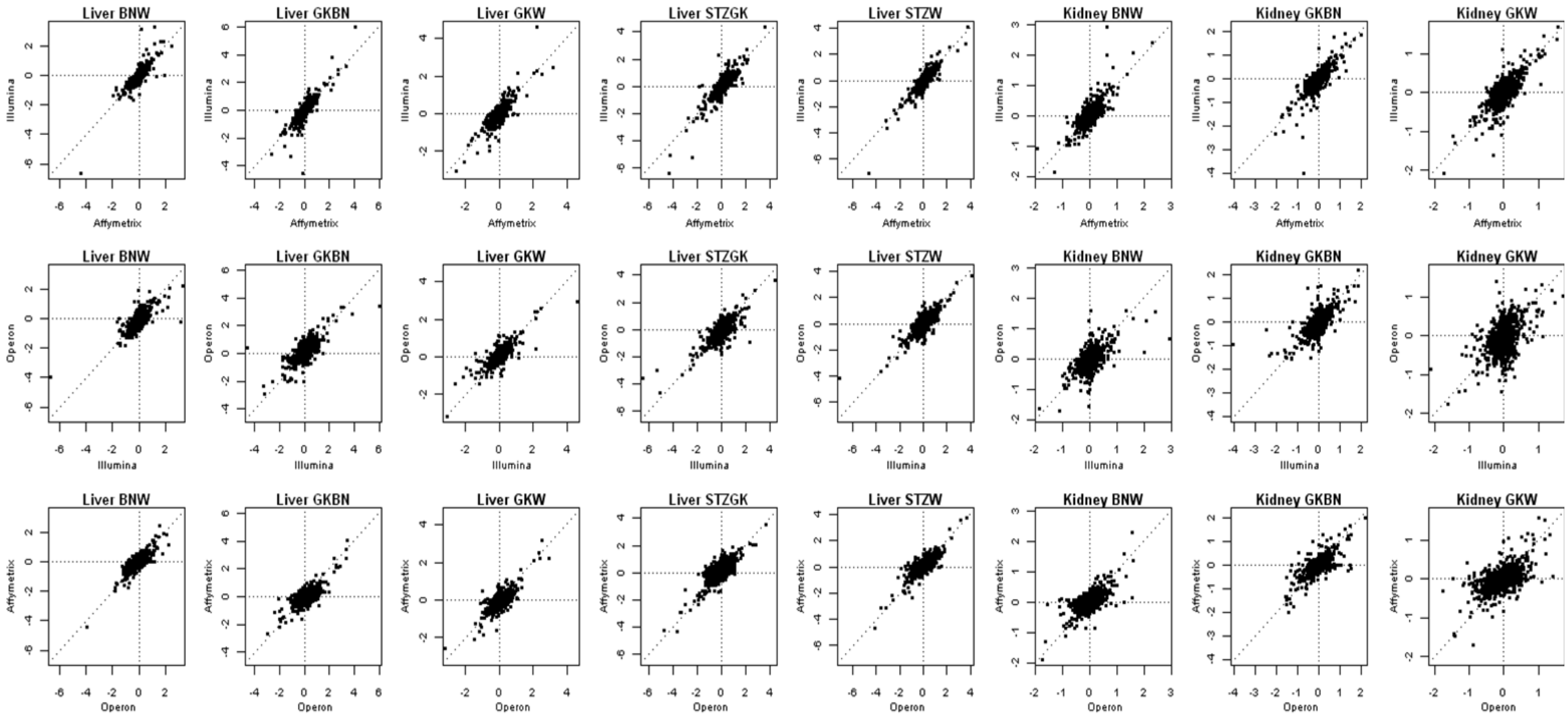

Supplement: Additional file 2 — Scatterplots of all rat Ensembl Gene match log2 fold changes between Affymetrix (normalised by RMA), Illumina (normalised by loess for liver and quantile normalisation for kidney) and Operon (normalised by vsn and quantile normalisation) for all tissues and strain comparisons. Analysis of rat gene expression changes for genes represented on the Illumina, Affymetrix and Operon arrays. [file 1471-2164-10-63-S2.pdf]
