# Supplementary material for: Comparative analysis of methods for gene transcription profiling data derived from different microarray technologies in rat and mouse models of diabetes
Source: BMC Genomics. 2009 Feb 5;10:63. doi: 10.1186/1471-2164-10-63 (PMC2652496; doi:10.1186/1471-2164-10-63)
Supplement: Additional file 4 — Gradient of log2 fold change scatterplots of Illumina (y) against Affymetrix (x) mouse "Target matches" for all normalisations, restricting analysis to the 25% most intense oligonucleotides (approximately 2,700 oligonucleotides). Effects of various normalisation methods on mouse gene expression changes generated by Illumina and Affymetrix arrays. [file 1471-2164-10-63-S4.pdf]

Additional file 4. Gradient of log2 fold change scatterplots of Illumina (*y)* against Affymetrix (*x*) mouse "Target matches" for all normalisations, restricting analysis to the 25% most intense oligonucleotides (approximately 2,700 oligonucleotides).

		Illumina normalisations					
		Scale	Quantile	Loess	Spline	Rank	vsn
Affymetrix normalisations	Scale – Avgdiff	0.99	0.98	0.97	1.05	0.99	1.14
	Scale – median polish	1.07	1.06	1.05	1.14	1.07	1.23
	Quantile – median polish	1.09	1.09	1.08	1.17	1.10	1.26
	Loess – median polish	1.09	1.09	1.09	1.16	1.10	1.26
	Spline – median polish	1.09	1.08	1.07	1.16	1.09	1.25
	MAS 5.0	0.58	0.57	0.57	0.62	0.58	0.68
	Li-Wong	0.65	0.65	0.65	0.70	0.66	0.76
	RMA	0.65	0.64	0.64	0.70	0.65	0.76
	GC-RMA	0.58	0.58	0.57	0.62	0.58	0.68
	vsn	1.01	1.00	1.00	1.08	1.02	1.17
