# Supplementary material for: Comparative analysis of methods for gene transcription profiling data derived from different microarray technologies in rat and mouse models of diabetes
Source: BMC Genomics. 2009 Feb 5;10:63. doi: 10.1186/1471-2164-10-63 (PMC2652496; doi:10.1186/1471-2164-10-63)
Supplement: Additional file 5 — Concordance of all mouse Affymetrix and quantile normalised Illumina unique "Target match" p value data between different Affymetrix normalisations (8,886 matches). Comparative analysis of statistical significance of mouse gene expression data derived by Illumina and Affymetrix arrays. [file 1471-2164-10-63-S5.pdf]

Additional file 5. Concordance of all mouse Affymetrix and quantile normalised Illumina unique "Target match" p value data between different Affymetrix normalisations (8,886 matches). The Illumina normalisation had a small effect relative to the choice for Affymetrix, so we only report quantile-normalised Illumina data.

	Affymetrix normalisations									
**Top**	Scale – Avgdiff	Scale – median polish	Quantile – median polish	Loess – median polish	Spline – median polish	MAS 5.0	Li-Wong	RMA	GC-RMA	vsn
**10**	5	4	4	5	3	6	5	5	6	5
**20**	7	6	9	9	7	8	10	11	9	10
**50**	21	22	24	25	22	18	26	26	19	24
**100**	37	41	38	40	37	36	44	43	37	38
**200**	80	74	86	87	75	65	78	86	84	87
**500**	220	233	238	226	218	140	199	231	227	241
**1000**	411	433	443	435	426	295	364	445	456	446
