# Supplementary material for: Comparative analysis of methods for gene transcription profiling data derived from different microarray technologies in rat and mouse models of diabetes
Source: BMC Genomics. 2009 Feb 5;10:63. doi: 10.1186/1471-2164-10-63 (PMC2652496; doi:10.1186/1471-2164-10-63)
Supplement: Additional file 6 — Concordance of all mouse Affymetrix and quantile normalised Illumina unique "Target match" fold change data between different Affymetrix normalisations (8,886 matches). Comparative analysis of the magnitude of mouse gene expression changes derived by Illumina and Affymetrix arrays. [file 1471-2164-10-63-S6.pdf]

Additional file 6. Concordance of all mouse Affymetrix and quantile normalised Illumina unique "Target match" fold change data between different Affymetrix normalisations (8,886 matches). The Illumina normalisation had a small effect relative to the choice for Affymetrix, so we only report quantile-normalised Illumina data.

	**Affymetrix normalisations**									
**Top**	Scale – Avgdiff	Scale – median polish	Quantile – median polish	Loess – median polish	Spline – median polish	MAS 5.0	Li-Wong	RMA	GC-RMA	vsn
**10**	8	7	7	7	7	2	7	7	8	7
**20**	12	13	14	14	14	6	12	17	17	15
**50**	23	25	28	29	27	7	20	35	34	28
**100**	56	58	64	61	61	10	34	67	67	66
**200**	102	104	120	120	112	17	64	129	129	122
**500**	236	247	282	278	267	38	103	283	290	287
**1000**	455	481	522	517	504	97	192	526	530	529
