# Supplementary material for: Comparative analysis of methods for gene transcription profiling data derived from different microarray technologies in rat and mouse models of diabetes
Source: BMC Genomics. 2009 Feb 5;10:63. doi: 10.1186/1471-2164-10-63 (PMC2652496; doi:10.1186/1471-2164-10-63)
Supplement: Additional file 7 — Total concordance in top X fold change lists between Affymetrix (normalised by GC-RMA), Illumina (normalised by loess for liver data and quantile for kidney data) and Operon (vsn and scale) for both tissues and all comparisons, using unfiltered (1,804 genes) and top 25% intensity-based filtering (approximately 280 genes). Comparative analysis of the magnitude of rat gene expression changes derived by Illumina, Affymetrix and Operon arrays. [file 1471-2164-10-63-S7.pdf]

Additional file 7. Total concordance in top *X* fold change lists between Affymetrix (normalised by GC-RMA), Illumina (normalised by loess for liver data and quantile for kidney data) and Operon (vsn and scale) for both tissues and all comparisons, using unfiltered (1,804 genes) and top 25% intensity-based filtering (approximately 280 genes).

			**Top**					
			**10**	**20**	**50**	**100**	**200**	**500**
**Liver**	**100%**	BNvWKY	5	9	26	44	84	217
		GKvBN	7	11	23	36	73	222
		GKvWKY	6	11	24	45	87	208
		STZvGK	6	14	25	46	85	232
		STZvWKY	9	12	29	48	82	218
	**25%**	BNvWKY	4	12	23	53	125	-
		GKvBN	7	12	19	47	128	-
		GKvWKY	7	11	28	56	130	-
		STZvGK	8	13	29	59	132	-
		STZvWKY	8	13	31	60	125	-
**Kidney**	**100%**	BNvWKY	4	7	18	30	59	177
		GKvBN	3	10	21	44	89	223
		GKvWKY	4	6	15	32	54	163
	**25%**	BNvWKY	4	11	20	43	116	-
		GKvBN	7	12	31	59	132	-
		GKvWKY	5	9	22	45	118	-
