# Supplementary material for: Comparative analysis of methods for gene transcription profiling data derived from different microarray technologies in rat and mouse models of diabetes
Source: BMC Genomics. 2009 Feb 5;10:63. doi: 10.1186/1471-2164-10-63 (PMC2652496; doi:10.1186/1471-2164-10-63)
Supplement: Additional file 8 — Total concordance in top X p value lists between Affymetrix (normalised by scale and median polish), Illumina (loess) and Operon (vsn and scale) for both tissues and all comparisons, using unfiltered (1,502 genes) and top 25% intensity-based filtering (approximately 280 genes). Comparative analysis of the statistical significance of rat gene expression changes derived by Illumina, Affymetrix and Operon arrays. [file 1471-2164-10-63-S8.pdf]

Additional file 8. Total concordance in top *X* p value lists between Affymetrix (normalised by scale and median polish), Illumina (loess) and Operon (vsn and scale) for both tissues and all comparisons, using unfiltered (1,502 genes) and top 25% intensity-based filtering (approximately 280 genes).

			**Top**					
			**10**	**20**	**50**	**100**	**200**	**500**
**Liver**	**100%**	BNvWKY	1	7	21	37	76	211
		GKvBN	2	8	18	34	65	229
		GKvWKY	3	10	19	44	81	219
		STZvGK	5	9	23	45	81	222
		STZvWKY	5	10	24	48	84	218
	**25%**	BNvWKY	5	9	21	46	122	-
		GKvBN	3	12	20	43	120	-
		GKvWKY	6	11	26	51	135	-
		STZvGK	6	11	28	58	129	-
		STZvWKY	5	11	29	52	127	-
**Kidney**	**100%**	BNvWKY	2	6	18	28	65	173
		GKvBN	1	5	22	44	80	196
		GKvWKY	3	5	14	27	52	163
	**25%**	BNvWKY	4	11	21	44	120	-
		GKvBN	3	10	30	57	126	-
		GKvWKY	2	6	16	40	121	-
