# Supplementary material for: Comparative analysis of methods for gene transcription profiling data derived from different microarray technologies in rat and mouse models of diabetes
Source: BMC Genomics. 2009 Feb 5;10:63. doi: 10.1186/1471-2164-10-63 (PMC2652496; doi:10.1186/1471-2164-10-63)

Additional file 9. Correlation in log2 fold change between qRT-PCR and all Affymetrix normalisations by strain comparison for all seventeen genes in rat kidney.

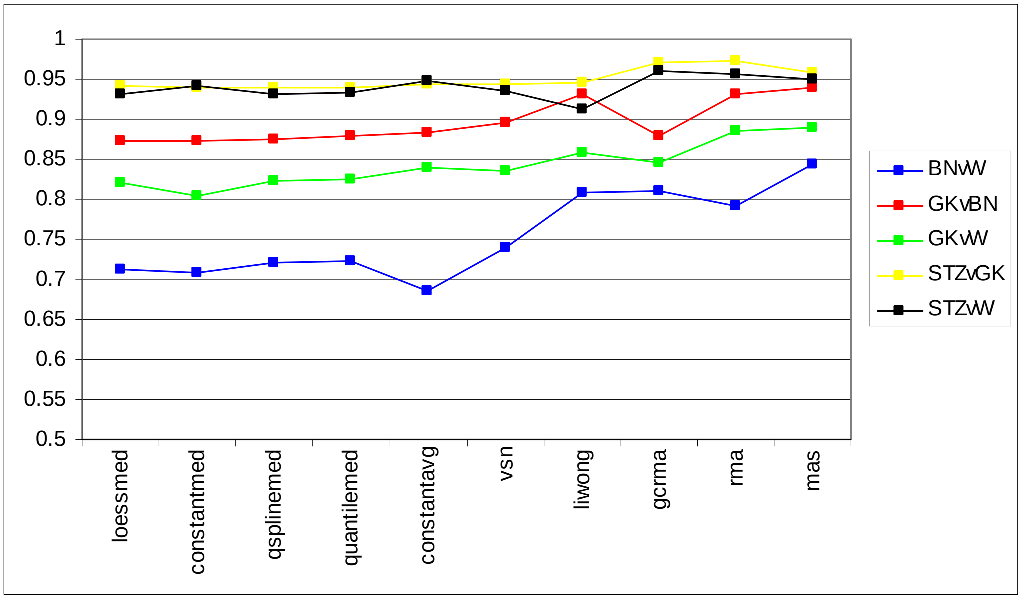

Supplement: Additional file 9 — Correlation in log2 fold change between qRT-PCR and all Affymetrix normalisations by strain comparison for all seventeen genes in rat kidney. Comparative analysis of gene expression changes given by quantitative RT-PCR and Affymetrix array data normalised using several methods. [file 1471-2164-10-63-S9.pdf]
