# Supplementary material for: Comparative analysis of methods for gene transcription profiling data derived from different microarray technologies in rat and mouse models of diabetes
Source: BMC Genomics. 2009 Feb 5;10:63. doi: 10.1186/1471-2164-10-63 (PMC2652496; doi:10.1186/1471-2164-10-63)
Supplement: Additional file 12 — Descriptions and log2 fold changes for all seventeen genes selected for qRT-PCR in all strain comparisons for the rat kidney experiment. Gene expression ratios derived by quantitative RT-PCR of renal samples from diabetic and control rats. [file 1471-2164-10-63-S12.pdf]

Additional file 12. Descriptions and log2 fold changes for all seventeen genes selected for qRT-PCR in all strain comparisons for the rat kidney experiment.

**Gene Symbol**	**Gene description**	**BNvWKY**	**GKvBN**	**GKvW**	**STZvGK**	**STZvWKY**
Ctgf	Connective tissue growth factor precursor	-0.7	2.3	1.6	-1.8	-0.2
Scd2	Acyl-CoA desaturase 2	0.5	-1.7	-1.2	1.7	0.5
Bmp6	Bone morphogenetic protein 6	-0.2	0.9	0.7	0.0	0.7
Ccnd1	G1/S-specific cyclin-D1	-0.3	0.8	0.5	0.0	0.5
Slc2a2	Glucose transporter type 2, liver	-0.2	1.7	1.6	0.4	2.0
Arntl	Brain and muscle ARNT-like 1	-1.3	0.2	-1.2	-0.9	-2.1
Eif2ak1	Eukaryotic translation initiation factor 2-alpha	0.3	-0.4	-0.1	0.5	0.4
Jund	Jun D proto-oncogene	1.7	0.0	1.7	-1.5	0.2
Bmp3	Bone morphogenetic protein 3 precursor	-0.5	-1.4	-1.8	0.6	-1.3
Hrg	Histidine-rich glycoprotein	-0.3	-0.4	-0.7	2.6	1.9
Calb1	Calbindin	0.8	-1.3	-0.6	1.7	1.2
Cktsf1b1	Gremlin-1 precursor	0.4	0.5	0.9	1.0	1.9
Cldn5	Claudin-5	-0.1	0.1	0.0	0.0	0.0
Cldn16	Claudin-16	-0.4	0.3	-0.1	-0.5	-0.6
Pld1	Phospholipase D1	0.0	0.3	0.3	-0.5	-0.2
Rbp1	Retinol-binding protein I	-0.1	-0.2	-0.3	1.4	1.1
Tff3	Trefoil factor 3 precursor	-1.1	0.4	-0.7	-4.1	-4.7
