# Supplementary material for: Comparative analysis of methods for gene transcription profiling data derived from different microarray technologies in rat and mouse models of diabetes
Source: BMC Genomics. 2009 Feb 5;10:63. doi: 10.1186/1471-2164-10-63 (PMC2652496; doi:10.1186/1471-2164-10-63)
Supplement: Additional file 13 — Gradient of log2 fold change fit of qRT-PCR (y) against microarray (x) for seventeen genes for selected normalisations for all microarray platforms for all rat kidney comparisons. Comparative analysis of renal gene expression ratios in rat models of diabetes and controls given by quantitative RT-PCR and normalised data from the corresponding genes on Illumina, Affymetrix and Operon arrays. [file 1471-2164-10-63-S13.pdf]

Additional file 13. Gradient of log2 fold change fit of qRT-PCR (*y*) against microarray (*x*) for seventeen genes for selected normalisations for all microarray platforms for all rat kidney comparisons.

Platform	Normalisation	BNvWKY	GKvBN	GKvWKY	STZvGK	STZvWKY
**Affymetrix**	Scale - Avgdiff	1.59	1.50	1.88	1.45	1.63
	Quantile – median polish	1.86	1.58	1.88	1.53	1.65
	MAS 5.0	0.85	1.03	0.82	1.10	1.19
	Li-Wong	1.36	1.37	1.36	1.32	1.31
	RMA	1.44	1.29	1.37	1.32	1.37
	GC-RMA	1.03	1.01	0.88	1.02	1.01
	Vsn	1.71	1.46	1.71	1.51	1.65
**Illumina**	Scale	1.28	1.23	1.10	1.19	1.29
	Loess	1.35	1.29	1.17	1.25	1.34
	Quantile	1.25	1.18	1.10	1.14	1.23
	Rank	1.31	1.26	1.14	1.22	1.31
	Spline	1.09	1.05	0.94	1.01	1.09
	Vsn	1.23	1.21	1.05	1.17	1.26
**Operon**	Edwards-spline-scale	0.95	0.90	0.90	-	-
	Kooperberg-loess-quantile	0.91	0.96	0.90	-	-
	Normexp-printtip loess-quantile	0.99	0.97	0.94	-	-
	vsn-scale	0.92	0.91	0.85	-	-
